# High-Sensitive CRP Correlates With the Severity of Liver Steatosis and Fibrosis in Obese Patients With Metabolic Dysfunction Associated Fatty Liver Disease

**DOI:** 10.3389/fendo.2022.848937

**Published:** 2022-05-06

**Authors:** Cuiling Zhu, Dongdong Huang, Huihui Ma, Chunhua Qian, Hui You, Le Bu, Shen Qu

**Affiliations:** ^1^ Department of Endocrinology and Metabolism, Shanghai Tenth People’s Hospital, Tongji University School of Medicine, Shanghai, China; ^2^ National Metabolic Management Center, School of Medicine, Shanghai Tenth People’s Hospital of Tongji University, Shanghai, China; ^3^ Department of Respiratory Medicine, School of Medicine, Shanghai Pulmonary Hospital of Tongji University, Shanghai, China

**Keywords:** high-sensitive C-reactive protein, metabolic dysfunction associated fatty liver disease, hepatic fibrosis, inflammation, obesity

## Abstract

**Background:**

Metabolic dysfunction associated fatty liver disease (MAFLD) is the most common hepatopathy worldwide due to the obesity epidemic and is associated with chronic low-grade inflammation. High-sensitive C-reactive protein (hsCRP) as an inflammatory marker has been used in diagnosing MAFLD. However, the association between hsCRP and the severity of liver steatosis and fibrosis among obese patients with MAFLD remains to be elucidated.

**Objective:**

To explore the correlation of hsCRP with the severity of liver steatosis and fibrosis among Chinese obese patients with MAFLD.

**Methods:**

A total of 393 obese patients with mean BMI 34.8 ± 6.6 kg/m^2^ were selected and categorized as MAFLD and non-MAFLD groups. Anthropometric data, biochemical indices, and hsCRP were measured. The severity of hepatic steatosis and fibrosis was assessed using FibroScan. Multivariate logistic regression analysis was performed to determine the relationship between hsCRP and the risk of MAFLD and its disease severity.

**Results:**

Patients with MAFLD showed significantly elevated hsCRP levels and were more likely to have severe steatosis and fibrosis compared to those without MAFLD. The proportions of MAFLD, severe steatosis, and severe fibrosis were significantly increased across the hsCRP quartiles (*P*-trend = 0.004, 0.021, and 0.006, respectively). After multivariable adjustments, the adjusted ORs (AORs) and 95%CI for MAFLD were 1.00 (reference), 1.298 (0.587-2.872), 2.407 (1.002-5.781), and 2.637(1.073-6.482) (Q1-Q4, *P*-trend = 0.014). Likewise, the AORs (95%CI) for severe steatosis and severe fibrosis were remarkably increased with the increment of serum hsCRP quartiles (*P*-trend < 0.001, *P*-trend = 0.021, respectively).

**Conclusions:**

Elevated serum hsCRP levels were associated with increased risk of MAFLD among Chinese obese patients and correlated positively with the severity of liver steatosis and fibrosis, suggesting that hsCRP can be used as a potential biomarker to monitor and predict disease severity among Chinese obese population with MAFLD.

## Introduction

Metabolic dysfunction associated fatty liver disease (MAFLD), the consensus-driven proposed nomenclature for non-alcoholic fatty liver disease (NAFLD) (1), has currently reached a worldwide epidemic, affecting approximately 25% of the overall population (2). It is increasing in its prevalence due to the obesity epidemic and thus poses enormous health and economic burden globally (3). It is reported that almost 80% of patients with MAFLD are accompanied by obesity that is believed to carry a higher risk for developing fibrosis and cirrhosis (4). MAFLD encompasses a spectrum of liver conditions ranging from simple steatosis to steatohepatitis, fibrosis, and cirrhosis (5). The pathogenic process of MAFLD is well−thought−out as a systemic metabolic dysfunction state (6) and commonly linked to various metabolic disorders, including fibrosis and cirrhosis (7), liver cancer (8), obesity (9), type 2 diabetes (T2DM) (10, 11), and even cardiovascular disease (12). It is worth noting that the clinical management and consequence of MAFLD patients are influenced strongly by the more aggressive phenotypes. For instance, fibrosis is the major determinant of adverse outcomes in patients with MAFLD, which could ultimately lead to liver cirrhosis, hepatocellular cancer, and death (13). Hence, early and accurate identification of patients with significant fibrosis and its possible pathogenesis among obese patients with MAFLD are crucial for clinicians to make the right therapeutic decisions and predict clinical outcomes.

Although the pathophysiology of MAFLD has been extensively studied, there is still a lot to uncover, especially in obese patients. Previous studies indicate that chronic low-grade inflammation occurs as a consequence of obesity (14) whereas recent insights suggest that it may play a causative role in generating insulin resistance (IR), insulin secretion deficiency, and imbalance of energy homeostasis (15). Of note, obesity-induced low-grade inflammation in the liver has been proposed as a possible contributory mechanism of MAFLD (16). High-sensitive C-reactive protein (hsCRP) as an inflammatory marker has been recently reported to be associated with MAFLD. Kumar et al. (17) reported that hsCRP levels were significantly higher in individuals with NAFLD and positively correlated with the disease severity of NAFLD in north Indian population. Kogiso et al. (18) also revealed that hsCRP was a strong predictor of the disease progression in NAFLD in the general Japanese population. Additionally, Yoneda et al. (19) showed significantly elevated hsCRP levels in patients with advanced fibrosis compared to those with mild fibrosis. Inconsistently, Zimmermann et al. (20) demonstrated a positive association of hsCRP with the degree of steatosis but not with the severity of NAFLD among obese patients in France. Collectively, the correlation between hsCRP and the disease progression of MAFLD might be complex based on different genetic background and study population.

Although hsCRP has been used clinically as an inflammatory marker in the diagnosis of MAFLD, little is known regarding its role in the disease progression of MAFLD among the Chinese obese population. In this work, we investigated the correlation of serum hsCRP with the risk of MAFLD and the severity of hepatic steatosis and fibrosis among the Chinese obese population, aiming to gain novel insights into the development and the disease progression of MAFLD.

## Methods

### Study Design and Participants

**Figure 1:**
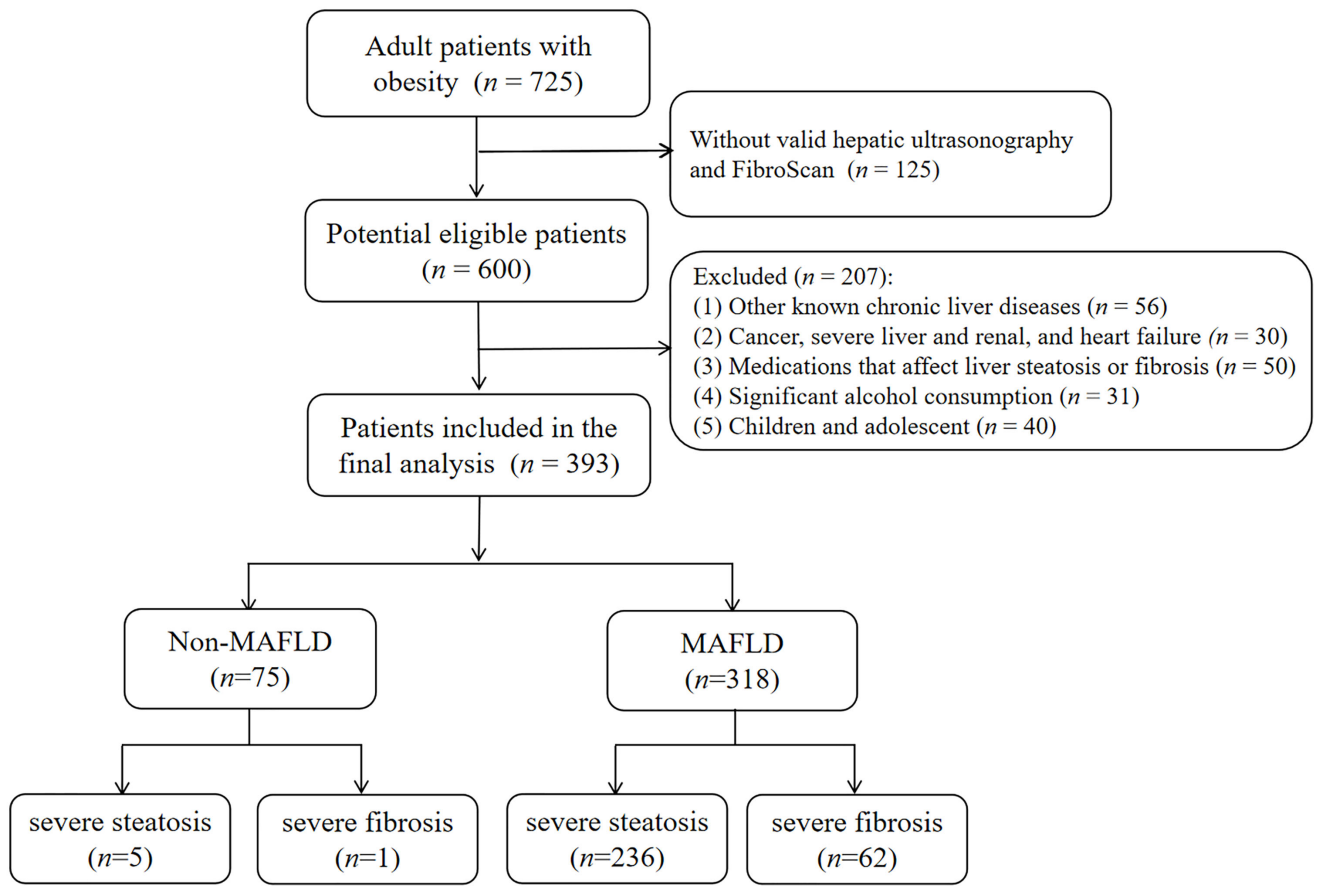
Flowchart of study enrollment. Of 725 adult obese patients, those who did not meet inclusion criteria (*n* =207) were excluded. Another 125 patients without valid hepatic ultrasonography and FibroScan were also excluded. As a result, 393 patients were included in the final analysis.

This retrospective cross-sectional study was conducted at the Department of Endocrinology and Metabolism, Shanghai Tenth People’s Hospital in China between January 2017 and October 2021. Initially, 725 adult obese patients who were admitted to our hospital were recruited. As a result, 393 subjects were finally included and divided into a MAFLD group (*n* = 318) and a non-MAFLD group (*n* = 75) based on the inclusion and exclusion criteria. The inclusion criteria were as follows (1): aged 18~65 years old (2), body mass index (BMI) ≥ 28 kg/m^2^ according to the diagnostic criteria for obesity in a Chinese population (21), and (3) underwent the biochemical tests, hepatic ultrasonography, and valid transient elastography (FibroScan) examination. Exclusion criteria included the presence of (1) other known chronic liver diseases, such as chronic hepatitis B or C, autoimmune hepatitis (2), pre-existing cancer, severe renal and liver dysfunction, and congestive heart disease (3), history of hyperthyroidism or hypothyroidism (4), significant alcohol consumption (22), and (5) pregnancy. Patients who were treated using any medication or other therapeutic methods that could influence liver steatosis or fibrosis, and glucolipid metabolism within 6 months prior to this study were also excluded. The flowchart of this study is shown in **Figure 1**.

### Clinical and Biochemical Measurements

Demographic and anthropometric data, including age, sex, height, body weight, systolic blood pressure (SBP), diastolic blood pressure (DBP), lifestyle factors (smoking status and alcohol consumption), comorbidities, and medical history were directly collected by trained physicians. Morning venous blood was drawn from all the participants after a 12-h overnight fast to measure the levels of alanine transaminase (ALT), aspartate aminotransferase (AST), gamma-glutamyl transferase (γ-GT), total cholesterol (TC), triglycerides (TG), low-density lipoprotein cholesterol (LDL-C), high-density lipoprotein cholesterol (HDL-C), fasting plasma glucose (FPG), and fasting insulin (FINS). Additionally, inflammation markers including interleukin (IL)-6, IL-8, tumor necrosis factor-α (TNF-α), and hsCRP levels were also measured. All the laboratory measurements were conducted in our department by the clinical laboratory using standard methodologies.

Herein, BMI was calculated as weight in kilograms divided by height in meters squared. Insulin resistance was calculated using the homeostasis model assessment of IR (HOMA-IR) as described by Matthews et al. (23): FPG (mmol/L) × FINS (mU/L)/22.5. According to the serum hsCRP levels, participants were divided into four quartiles (Q1, Q2, Q3, and Q4) as follows: Q1, <3.30 mg/L; Q2, 3.30~4.46 mg/L; Q3, 4.46~6.68 mg/L; and Q4, ≥ 6.68 mg/L.

### Definition of MAFLD

Patients were diagnosed as MAFLD based on evidence of ultrasonically diagnosed hepatic steatosis in addition to one of the following three criteria, namely overweight/obesity, T2DM, or metabolic dysregulation regardless of alcohol consumption or other concomitant liver diseases, which was proposed by the international expert consensus statement in 2020 (1). Herein, the metabolic dysregulation was defined by the presence of at least two metabolic risk abnormalities found in lean or normal weight patients, including hypertension, dyslipidemia, hyperglycemia, IR, and high CRP levels. Among which, hypertension was considered if their SBP ≥ 130 mmHg or DBP ≥ 85 mmHg or they were taking antihypertensive drugs. The presence of dyslipidemia was ascertained by plasma TG ≥ 1.7mmol/L in the total population or plasma HDL‐C < 1.0 mmol/L for men and < 1.3 mmol/L for women or was taking specific lipid-lowering drugs. T2DM was diagnosed according to the guideline for the prevention and treatment of type 2 diabetes mellitus in China (2020 edition) (24). In addition, plasma CRP > 2 mg/L or HOMA-IR ≥ 2.5 were also considered as MAFLD-associated metabolic abnormalities. As a result, 25 patients were stratified into the MAFLD group only based on ultrasonically diagnosed hepatic steatosis and high hsCRP levels. Patients who did not meet any of the above conditions were referred to as the non-MAFLD group.

### Liver Assessment and Subgroups

Hepatic steatosis in this study was determined by hepatic ultrasonography according to the guidelines for prevention and treatment of NAFLD (25). Controlled attenuation parameter (CAP) and liver stiffness measurement (LSM) were obtained from transient elastography (FibroScan^®^) by physicians trained and certified by the manufacturer. Only examinations with at least 10 valid individual measurements were deemed valid. Furthermore, we estimated the severity of liver steatosis and fibrosis according to results from FibroScan (26). Based on CAP values, liver steatosis was graded as follows: absent: < 302 dB/m, mild to moderate: 302-337 dB/m, and severe: ≥337 dB/m. Based on LSM values, hepatic fibrosis was staged as follows: absent: < 8.2 kPa, mild to moderate: 8.2-13.6 kPa, and severe: ≥13.6 kPa. All the hepatic ultrasonography and transient elastography were performed and evaluated by experienced ultrasonographers who were blinded to the participants’ clinical and biochemical details.

### Statistical Analysis

All statistical analyses were performed using SPSS 23.0 software (SPSS Inc., Chicago, IL) and the figures were produced by GraphPad Prism 6.0 project. Continuous variables were reported as means ± standard deviation (SD) or medians (interquartile ranges) based on data distribution and compared by independent Student’s t-test or one-way analysis of variance (ANOVA). Categorical variables were expressed as numbers and percentages and compared by chi-squared test. Non-normally distributed data were analyzed by non-parametric test and logarithmically transformed to normality when appropriate. The bivariate Pearson correlation analysis was carried out to determine the relationship between hsCRP and other related metabolic parameters. Univariable logistic regression analysis was conducted to explore the contribution of hsCRP and other risk factors in MAFLD and its progression. Multivariate logistic regression analysis was used to estimate the adjusted odds ratios (AORs) and 95% confidence intervals (CIs) for MAFLD, severe steatosis, and severe fibrosis in each hsCRP quartile in three regression models (1): for age, gender, and BMI (2); for age, gender, BMI, ALT, AST, and γ-GT; and (3) for TC, TG, HDL-C, LDL-C, LnFINS, LnHOMA-IR in addition to all covariates in (2). A two‐tailed P < 0.05 was considered to be statistically significant.

## Results

### Clinical Characteristics of Study Participants

In total, 393 obese patients (192 men and 201 women) with mean age 38.9 ± 16.6 years and mean BMI 34.8 ± 6.6 kg/m^2^ were analyzed in the present study. The prevalence of MAFLD, severe liver steatosis, and liver fibrosis in the cohort was 80.9% (*n* = 318), 61.3% (*n* = 241), and 16.0% (*n* = 63), respectively. Baseline clinical characteristics of the study population stratified by MAFLD status are depicted in **Table 1**. Compared to the non-MAFLD group, the MAFLD group tended to be younger (*P* < 0.001) and had significantly higher BMI, DBP, ALT, AST, γ-GT, TC, TG, LDL-C, LnFINS, and LnHOMA-IR, as well as lower HDL-C levels (all *P* < 0.05). Concerning inflammatory markers, serum hsCRP levels were remarkably elevated in the MAFLD group (5.9 ± 3.8 mg/L vs. 4.5 ± 2.2 mg/L, *P* < 0.001) as opposed to their counterparts whereas IL-6, IL-8, and TNF-α levels were unchanged between the two groups. This increased hsCRP level in the MAFLD group is at least partly due to the study design in that serum hsCRP was used as a criterion to stratify MAFLD. Additionally, the overall proportions of hypertension, hyperlipidemia, and T2DM in the MAFLD group were significantly higher than those in the non-MAFLD group (*P* = 0.004, *P* = 0.008, *P* = 0.021, respectively). According to the results from FibroScan, severe and mild to moderate liver steatosis were found in 236 (74.3%) and 82 (25.7%) patients in the MAFLD group, which was significantly higher than those in the non-MAFLD group (all *P <*0.001). Likely, the percentage of patients with severe and mild to moderate liver fibrosis was remarkably higher in the MAFLD group as opposed to those in the non-MAFLD group (severe: 19.6% vs. 1.3%, *P* < 0.001; mild-moderate: 46.8% vs. 24.0%, *P* < 0.001).

**Table 1:** Baseline characteristics of the study cohort stratified by MAFLD status.

Parameters	Total population	Non-MAFLD	MAFLD	*P* value
	*n* = 393	*n* = 75	*n* = 318	
**Demographics**				
Gender (male)	192 (48.8%)	35 (46.6%)	157 (49.4%)	0.674
Age (yr)	38.9 ± 16.6	47.2 ± 18.3	36.9 ± 15.6	<0.001
BMI (kg/m^2^)	34.8 ± 6.6	31.8 ± 5.4	35.4 ± 6.7	<0.001
SBP (mmHg)	137.0 ± 17.0	135.0 ± 18.0	137.0 ± 18.0	0.390
DBP (mmHg)	81.0 ± 14.0	76.0 ± 11.0	82.0 ± 14.0	0.006
**Laboratory parameters**				
ALT (U/L)	46.9 ± 40.3	29.6 ± 23.4	50.9 ± 42.3	<0.001
AST (U/L)	32.5 ± 22.4	24.5 ± 14.8	34.4 ± 23.5	<0.001
γ-GT (U/L)	44.4 ± 37.9	29.6 ± 19.4	47.4 ± 39.8	<0.001
TC (mmol/L)	4.6 ± 0.9	4.4 ± 0.9	4.7 ± 0.9	0.040
TG (mmol/L)	2.2 ± 1.3	1.7 ± 0.8	2.3 ± 1.4	<0.001
HDL-C (mmol/L)	1.1 ± 0.3	1.2 ± 0.6	1.0 ± 0.2	0.001
LDL-C (mmol/L)	2.8 ± 0.8	2.5 ± 0.8	2.8 ± 0.8	0.015
FPG (mmol/L)	7.4 ± 2.5	7.4 ± 2.5	7.4 ± 2.6	0.985
Ln FINS (mU/L)	3.0 ± 0.7	2.7 ± 0.7	3.1 ± 0.7	<0.001
Ln HOMA-IR	1.9 ± 0.7	1.6 ± 0.7	1.9 ± 0.7	<0.001
**Inflammatory markers**				
IL-6 (pg/ml)	5.7 (11.2)	4.1 (8.1)	5.8 (11.3)	0.054
IL-8 (pg/ml)	106.0 (462.4)	53.7 (356.6)	119.0 (474.7)	0.106
TNF-α (pg/ml)	12.2 (15.4)	8.6 (9.3)	12.9 (15.8)	0.263
hsCRP (mg/L)	5.6 ± 3.6	4.5 ± 2.2	5.9 ± 3.8	<0.001
**Comorbidities, *n* (%)**				
Hypertension	253 (65.9)	39 (52.0)	214 (67.3)	0.004
Hyperlipidemia	376 (95.7)	69 (89.6)	307 (97.2)	0.008
Type 2 diabetes	370 (75.2)	45 (64.3)	225 (77.9)	0.021
**Liver steatosis, *n* (%)**				
Absent	64 (16.3)	64 (85.5)	0 (0.0)	<0.001
Mild to moderate	88 (22.4)	6 (8.0)	82 (25.7)	<0.001
Severe steatosis	241 (61.3)	5 (6.5)	236 (74.3)	<0.001
**Liver fibrosis, *n* (%)**				
Absent	163 (41.5)	56 (74.6)	107 (33.6)	<0.001
Mild to moderate	167 (42.5)	18 (24.0)	149 (46.8)	<0.001
Severe fibrosis	63 (16.0)	1 (1.3)	62 (19.6)	<0.001

Continuous data are presented as means ± standard deviations (SD) or medians (interquartile ranges). Non-normally distributed data were log-transformed before analysis. Categorical variables are presented as percentages (%). BMI, body mass index; SBP, systolic blood pressure; DBP, diastolic blood pressure; ALT, alanine transaminase; AST, aspartate aminotransferase; γ-GT, gamma-glutamyl transferase; TC, total cholesterol; TG, triglyceride; HDL-C, high-density lipoprotein cholesterol; LDL-C, low-density lipoprotein cholesterol; FPG, fasting plasma glucose; FINS, fasting insulin; HOMA-IR, homeostasis model assessment of insulin resistance; hsCRP, high-sensitive C reactive protein; IL-6, interleukin-6; IL-8, interleukin-8; TNF-α, tumor necrosis factor-α. MAFLD vs. Non-MAFLD, P-values < 0.05 were accepted as statistically significant.

### Proportions of MAFLD, Severe Steatosis, and Fibrosis Across hsCRP Quartiles

**Figure 2:**
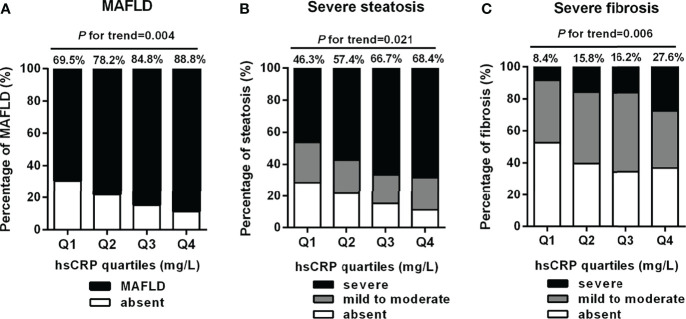
Percentage of MAFLD, severe steatosis, and severe fibrosis across serum hsCRP quartiles in obese patients. Serum hsCRP levels were plotted into four quartiles (Q1, Q2, Q3, and Q4). There was a significantly increasing trend in the percentage of MAFLD **(A)**, severe steatosis **(B)**, and severe fibrosis **(C)** across serum hsCRP quartiles (*P*-trend = 0.004, 0.021, and 0.006, respectively). *P*-values < 0.05 were accepted as statistically significant.

To explore the correlation of serum hsCRP with risk of MAFLD and its progression among obese subjects, we grouped patients into four groups (Q1, Q2, Q3, Q4) based on serum hsCRP levels. As shown in **Figure 2**, there was a significantly increasing trend in the percentage of MAFLD, severe steatosis, and severe fibrosis with the increment of serum hsCRP quartiles. The constituent ratio of MAFLD was 69.5% in Q1, 78.2% in Q2, 84.8% in Q3, and 88.8% in Q4, respectively (*P-*trend = 0.004) (**Figure 2A**). Accordingly, the proportion of severe steatosis was 46.3%, 57.4%, 66.7%, and 68.4% (Q1-Q4, *P-*trend = 0.021) (**Figure 2B**). Likewise, the proportion of severe fibrosis was significantly increased across the serum hsCRP quartiles (8.4%, 15.8%, 16.2%, and 27.6%, Q1-Q4, *P-*trend = 0.006) (**Figure 2C**).

### Indicators for MAFLD and Severe Steatosis and Fibrosis

The factors associated with the risk of MAFLD, severe steatosis, and fibrosis in obese patients are presented in **Table 2**. Among them, age, BMI, ALT, AST, γ-GT, TC, TG, HDL-C, LDL-C, FPG, LnFINS, and LnHOMA-IR were fitted in quartile categories. The category boundaries of each variable are described in the footnote of **Table 2**. The univariate logistic regression analysis demonstrated a significantly higher odds ratios (ORs) for MAFLD in obese patients with elevated BMI (ORs 1.637, 95%CI 1.183-2.266, *P* = 0.003), γ-GT (ORs 1.500, 95%CI 1.068-2.107, *P* = 0.019), LnHOMA-IR (ORs 1.195, 95%CI 1.076-1.842, *P* = 0.009), and hsCRP (ORs 1.203, 95%CI 1.065-2.358, *P* = 0.003). In addition, the ORs for severe steatosis were significantly increased among obese participants with women (ORs 2.093, 95%CI 1.222-2.583, *P* < 0.001), higher BMI (ORs 1.824, 95%CI 1.405-2.367, *P* < 0.001), γ-GT (ORs 1.342, 95%CI 1.018-1.768, *P* = 0.037), LDL-C (ORs 1.426, 95%CI 1.040-1.955, *P* = 0.027), LnHOMA-IR (ORs 1.140, 95%CI 1.060-1.338, *P* = 0.030), and hsCRP (ORs 1.159, 95%CI 1.065-2.262, *P* = 0.001). Similarly, there was a remarkably elevated risk for severe fibrosis among obese patients with higher BMI (ORs 1.488, 95%CI 1.044-2.119, *P* = 0.028), TC (ORs 1.567, 95%CI 1.126-1.986, *P* = 0.044), TG (ORs 1.582, 95%CI 1.128-2.220, *P* = 0.008), LnHOMA-IR (ORs 1.178, 95%CI 1.073-1.346, *P* = 0.010), and hsCRP (ORs 1.163, 95%CI 1.078-1.254, *P* < 0.001). Conversely, age (ORs 0.678, 95%CI 0.468-0.981, *P* = 0.039) was inversely associated with severe fibrosis.

**Table 2:** Odds ratios (ORs) of multiple variables for MAFLD, severe steatosis, and severe fibrosis.

Variables	MAFLD			Severe steatosis			Severe fibrosis		
	ORs	95%CI	*P* value	ORs	95%CI	*P* value	ORs	95%CI	*P* value
Gender	1.532	0.788, 2.980	0.208	2.093	1.222, 2.583	0.007	1.195	0.597, 2.394	0.615
Age quartiles	0.951	0.680, 1.329	0.767	0.983	0.748, 1.292	0.903	0.678	0.468, 0.981	0.039
BMI quartiles	1.637	1.183, 2.266	0.003	1.824	1.405, 2.367	<0.001	1.488	1.044, 2.119	0.028
CRP	1.203	1.065, 2.358	0.003	1.159	1.065, 1.262	0.001	1.163	1.078, 1.254	<0.001
ALT quartiles	1.417	0.924, 2.173	0.110	1.294	0.914, 1.831	0.146	0.995	0.633, 1.565	0.984
AST quartiles	0.811	0.541, 1.214	0.308	0.758	0.544, 1.055	0.101	0.906	0.596, 1.379	0.646
γ-GT quartiles	1.500	1.068, 2.107	0.019	1.342	1.018, 1.768	0.037	1.219	0.833, 1.785	0.308
TC quartiles	0.807	0.537, 1.214	0.304	0.805	0.568, 1.143	0.225	1.567	1.126, 1.986	0.044
TG quartiles	1.119	0.813, 1.541	0.489	0.971	0.748, 1.261	0.825	1.582	1.128, 2.220	0.008
HDL-C quartiles	0.859	0.642, 1.147	0.303	1.006	0.794, 1.274	0.961	1.176	0.860, 1.606	0.310
LDL-C quartiles	1.305	0.902, 1.888	0.158	1.426	1.040, 1.955	0.027	1.565	0.949, 2.582	0.080
FPG quartiles	1.025	0.752, 1.397	0.875	0.993	0.773, 1.277	0.959	0.827	0.599, 1.142	0.250
LnFINS quartiles	0.911	0.588, 1.412	0.678	1.032	0.720, 1.479	0.863	1.406	0.891, 2.218	0.143
LnHOMAIR quartiles	1.532	0.788, 2.980	0.208	2.093	1.222, 2.583	0.007	1.195	0.597, 2.394	0.615

ORs were determined by univariable logistic regression analysis. Age was plotted in quartiles with value set at < 26, 26-35, 35-54, and ≧54 years old. BMI was plotted in quartiles with levels set at < 29.1, 29.1-33.3, 33.3-38.5, and ≧38.5kg/m^2^. ALT was plotted in quartiles with levels set at <20.6, 20.6-33.9, 33.9-63.9, and ≧63.9 U/L. AST was plotted in quartiles with levels set at <17.7, 17.7-25.9, 25.9-39.5, and ≧39.5 U/L. γ-GT was plotted in quartiles with levels set at < 23.1, 23.1-35.8, 35.8-55.3, and ≧55.3 U/L. TC was plotted in quartiles with levels set at < 4.04, 4.04-4.61, 4.61-5.30, and ≧5.30 mmol/L. TG was plotted in quartiles with levels set at <1.3, 1.3-1.8, 1.80-2.80, and ≧2.80 mmol/L. HDL-C was plotted in quartiles with levels set at <0.89, 0.89-1.04, 1.04-1.19, and ≧1.19 mmol/L. LDL-C was plotted in quartiles with levels set at <2.1, 2.1-2.7, 2.7-3.4, and ≧3.4 mmol/L. FPG was plotted in quartiles with levels set at <5.6, 5.6-7.0, 7.0-8.2, and ≧8.2 mmol/L. LnFINS was plotted in quartiles with levels set at < 2.5, 2.5-3.1, 3.1-3.6, and ≧3.6 mU/L. LnHOMA-IR was plotted in quartiles with levels set at < 1.4, 1.4-1.9, 1.9-2.4, and ≧2.4. P-values < 0.05 were accepted as statistically significant.

**Figure 3:**
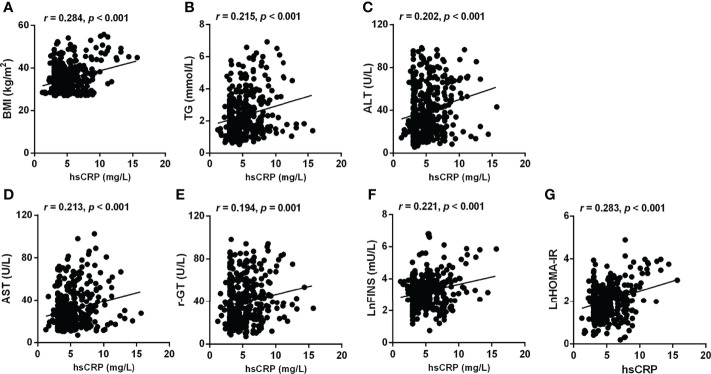
Correlations between hsCRP and other metabolic risk factors related to MAFLD, steatosis, and fibrosis. Serum hsCRP levels were positively associated with BMI **(A)**, TG **(B)**, ALT **(C)**, AST **(D)**, γ-GT **(E)**, LnFINS **(F)**, and LnHOMA-IR **(G)**. Non-normally distributed data were log-transformed before analysis. *P*-values < 0.05 were accepted as statistically significant.

In the bivariate correlation analysis, serum hsCRP was positively associated with BMI (*r* = 0.284, *P* < 0.001), TG (*r* = 0.215, *P* < 0.001), ALT (*r* = 0.202, *P* < 0.001), AST (*r* = 0.213, *P* < 0.001), γ-GT (*r* = 0.194, *P* = 0.001), LnFINS (*r* = 0.221, *P* < 0.001), and LnHOMA-IR (*r* = 0.283, *P* < 0.001) (**Figure 3**). However, no significant correlation was observed between hsCRP and age, TC, HDL-C, LDL-C, and FPG.

### Relationship Between hsCRP, MAFLD, and the Severity of Liver Steatosis and Fibrosis

**Figure 4:**
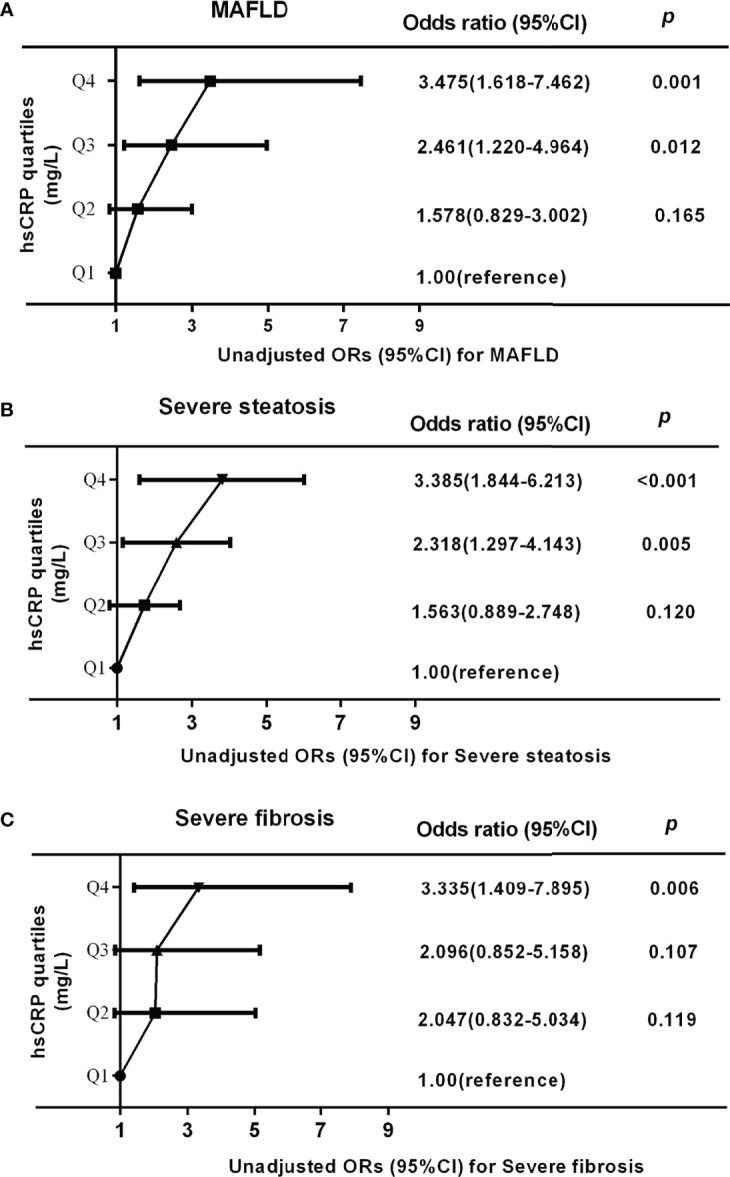
Unadjusted odds ratios (ORs) and 95% confidence intervals (CI) for MAFLD, severe steatosis, and severe fibrosis in obese patients according to serum hsCRP quartiles: results of binary logistic regression analysis. Serum hsCRP levels were plotted into four quartiles (Q1, Q2, Q3, and Q4). **(A, B)** The ORs (95%CI) for MAFLD and severe steatosis in Q3 and Q4 quartile were significantly higher than that in Q1 quartile (all *P-*trend < 0.001). **(C)** The ORs (95%CI) for severe fibrosis in Q4 quartile were significantly higher than that in Q1 quartile (*P*-trend = 0.007). *P*-values < 0.05 were accepted as statistically significant.

Binary logistic regression analysis was applied to further delineate the relationship between hsCRP and MAFLD and its disease progression based on hsCRP serum quartiles. In the univariate logistic regression analysis, the unadjusted ORs (95%CI) for MAFLD were significantly higher in Q3 (ORs 2.461, 95%CI 1.220-4.964, *P* = 0.012) and Q4 (ORs 3.475, 95%CI 1.618-7.462, *P* = 0.001) than that in Q1 (as reference) (**Figure 4A**). A similar result was found in the correlation between hsCRP and severe steatosis (**Figure 4B**). Furthermore, there was a dramatically elevated risk for severe fibrosis in Q4 (ORs 3.335, 95%CI 1.409-7.895, *P* = 0.006) compared to Q1 (**Figure 4C**). After adjusting for potential confounders, the adjusted ORs (AORs) and 95%CI for MAFLD, severe steatosis, and severe fibrosis remained increased across the hsCRP quartiles in three models (age, gender, and BMI involved in Model 1; age, gender, BMI, ALT, AST, and γ-GT involved in Model 2; age, gender, BMI, ALT, AST, γ-GT, TC, TG, HDL-C, LDL-C, LnFINS, and LnHOMA-IR involved in Model 3) (**Table 3**). The *P* for trend for MAFLD was 0.001 in Model 1, 0.006 in Model 2, and 0.014 in Model 3. Also, the *P* for trend for severe steatosis (all *P*-trend < 0.001) and fibrosis (all *P*-trend < 0.05) was significant in all three models.

**Table 3:** Adjusted ORs and 95%CI for MAFLD, severe steatosis, and severe fibrosis based on hsCRP quartiles: results of binary logistic regression analysis.

hsCRP quartiles (mg/L)	For MAFLD			For severe steatosis			For severe fibrosis		
	β	Adjusted OR (95%CI)	*P*	β	Adjusted OR (95%CI)	*P*	β	Adjusted OR (95%CI)	*P*
**Model 1**									
Q1 (<3.30)		Ref	0.014		Ref	0.001			0.079
Q2 (3.30-4.66)	0.402	1.494 (0.755-2.957)	0.249	0.354	1.424 (0.778-2.607)	0.252	0.566	1.761 (0.690-4.495)	0.237
Q3 (4.66-6.68)	0.786	2.195 (1.053-4.576)	0.036	0.774	2.169 (1.170-4.018)	0.014	0.578	1.782 ().701-4.528)	0.225
Q4 (≥6.68)	1.241	3.460 (1.556-7.697)	0.002	1.303	3.681 (1.914-7.076)	<0.001	1.169	3.218 (1.297-7.983)	0.012
*P* for trend	0.001			<0.001			0.014		
**Model 2**									
Q1 (<3.30)		Ref	0.050		Ref	0.001		Ref	0.131
Q2 (3.30-4.66)	0.213	1.238 (0.596-2.569)	0.567	0.387	1.473 (0.789-2.752)	0.224	0.647	1.910 (0.738-4.941)	0.182
Q3 (4.66-6.68)	0.805	2.237 (1.008-4.965)	0.048	0.816	2.261 (1.199-4.262)	0.012	0.608	1.837 (0.718-4.697)	0.204
Q4 (≥6.68)	1.046	2.847 (1.203-6.735)	0.017	1.340	3.818 (1.946-7.490)	<0.001	1.109	3.030 (1.203-7.633)	0.019
*P* for trend	0.006			<0.001			0.026		
**Model 3**									
Q1 (<3.30)		Ref	0.093		Ref	0.001			0.119
Q2 (3.30-4.66)	0.261	1.298 (0.587-2.872)	0.520	0.273	1.314 (0.677-2.550)	0.420	0.830	2.293 (0.818-6.422)	0.114
Q3 (4.66-6.68)	0.878	2.407 (1.002-5.781)	0.049	0.838	2.312 (1.170-4.569)	0.016	0.441	1.554 (0.547-4.414)	0.408
Q4 (≥6.68)	0.970	2.637 (1.073-6.482)	0.035	1.300	3.670 (1.832-7.353)	<0.001	1.127	3.086 (1.154-8.249)	0.025
*P* for trend	0.014			<0.001			0.021		

Serum hsCRP levels were plotted into four quartiles (Q1, Q2, Q3, and Q4). Model 1: age, gender, and BMI were selected. Model 2: age, gender, BMI, ALT, AST, and γ-GT were selected. Model 3: age, gender, BMI, ALT, AST, γ-GT, TC, TG, HDL-C, LDL-C, LnFINS, and LnHOMA-IR were selected. P-values <0.05 were accepted as statistically significant.

## Discussion

In the present study, serum hsCRP levels were strongly correlated with the risk of MAFLD and the severity of hepatic steatosis and fibrosis among the Chinese obese population. These results remained significant even after adjusting potential confounding factors, indicating that hsCRP may be an independent risk factor regarding the progression of MAFLD into more aggressive phenotypes. These associations may be mediated by substantial IR and inflammation status in addition to excess weight, dyslipidemia, and increased liver enzymes. Hence, our findings provide a new insight into the correlation between serum hsCRP levels and the occurrence of MAFLD, as well as the severity of liver steatosis and fibrosis. It raises the possibility that hsCRP can be used as a potential biomarker for the disease severity in MAFLD among the Chinese obese population or, at least, identify therapeutic approaches to modulate inflammation in the context of obesity and MAFLD.

MAFLD, formerly named NAFLD, is a new definition of liver disease associated with known metabolic dysfunction (1), and is commonly associated with various metabolic disorders including obesity, IR, T2DM, dyslipidemia, elevated liver enzymes, and inflammatory markers (11, 27–30). It has also been reported to identify individuals with liver steatosis and significant fibrosis better than NAFLD (7). In agreement with this, the current study showed that patients with MAFLD had significantly higher BMI, ALT, AST, γ-GT, TC, TG, LDL-C, LnFINS, and LnHOMA-IR, as well as lower HDL-C levels compared to those without MAFLD. In addition, the proportions of hypertension, hyperlipidemia, T2DM, and severe hepatic steatosis and fibrosis were remarkably increased in the MAFLD group instead of the non-MAFLD group. Based on these complications accompanied by MAFLD, it is extremely crucial for us to determine key factors and potential mechanisms of MAFLD and identify individuals with severe liver steatosis and fibrosis, aiming to provide clinical evidence for prevention and treatment within a similar population.

Obesity-induced chronic low-grade inflammation has been documented to play a fundamental role in the pathogenesis of MAFLD (31). As one of the inflammatory markers, hsCRP has been previously recommended to help diagnose NAFLD or NASH. Yeniova et al. (32) demonstrated that hsCRP levels were significantly higher in patients with NAFLD compared to their counterparts. In one case-control study among Asian Indians in North India, higher hsCRP levels were correlated with increased risk of NAFLD (33). In another case-control study including 32 cases with biopsy-proven NAFLD and 34 non-obese control subjects, serum hsCRP levels were found to be higher in the NASH group compared to its control group among NAFLD cases, and considerably increased in higher fibrosis stage (34). Consistently, Yoneda et al. (19) revealed marked elevated hsCRP levels in cases with NASH than in those with simple steatosis. Also, elevated hsCRP levels were correlated with increased risk for advanced fibrosis in the same study, suggesting that serum hsCRP may be a useful non-invasive marker that not only differentiates between NASH and simple steatosis in NAFLD patients, but also predicts the severity of hepatic fibrosis in cases with NASH. In disagreement with this, one study including 627 obese adults showed a positive correlation of hsCRP with degree of steatosis but not with severity of NAFLD (20). In contrast with NAFLD, MAFLD is a multisystem metabolic disease with higher proportions of metabolic comorbidities, supporting that MAFLD is more practical for identifying patients with fatty liver disease with high risk of disease progression (35). However, there is scarce evidence regarding the association between hsCRP and the risk of MAFLD especially among Chinese obese patients. In the present study, we demonstrated that serum hsCRP levels were significantly higher in individuals with MAFLD than in those without MAFLD among Chinese obese patients. In addition, the proportion of MAFLD was significantly increased with the increment of serum hsCRP quartiles. Furthermore, we observed a significantly increased risk for MAFLD across the hsCRP quartiles even after adjusting for potential confounding factors (age, gender, BMI, ALT, AST, γ-GT, TC, TG, HDL-C, LDL-C, LnFINS, and LnHOMA-IR). These results indicate that serum hsCRP might be an independent risk factor of MAFLD among Chinese obese patients.

Accumulating evidence has validated that hepatic fibrosis is the major adverse outcome in patients with MAFLD (13). Although serum hsCRP has been confirmed previously to have significant impact on the NAFLD pathophysiology and can be used clinically as an inflammatory marker in diagnosing MAFLD in lean or normal weight patients, extremely limited studies were done regarding the influence of serum hsCRP on the disease progression of MAFLD. It is worth noting that CAP and LSM obtained from transient elastography (FibroScan^®^) have been recommended as useful tools to detect both hepatic steatosis and fibrosis in MAFLD (26). In accordance with literature consensus (36), we assessed the severity of liver steatosis and fibrosis in MAFLD using FibroScan and investigated their correlation with serum hsCRP level. The results showed that the proportions of severe steatosis and fibrosis were increased significantly across serum hsCRP quartiles. Moreover, the binary logistic regression analysis demonstrated remarkably increased ORs (95%CI) for severe steatosis and fibrosis with the rise in serum hsCRP levels. This result remained statistically significant after adjustment for cardiometabolic risk factors (age, gender, BMI, ALT, AST, γ-GT, TC, TG, HDL-C, LDL-C, LnFINS, and LnHOMA-IR), which are widely held MAFLD determining factors. These findings implied that elevated serum hsCRP could be a potential biomarker to monitor the disease progression of MAFLD among Chinese obese patients. However, the previous evidence produced conflicting results. Some study reported that hsCRP levels were positively correlated with the grade of steatosis among Asian Indians (17), whereas another study demonstrated no association between hsCRP and the severity of steatosis and fibrosis in obese women (37). Another study showed that hsCRP was positively associated with degree of steatosis but not the severity of fibrosis in obese adults from France and Belgium (20). These discrepancies might be explained by differences in the race, dietary structure, and environment, as well as the genetic makeup of the study population which have been validated to be important predictors in the development of MAFLD (32).

It is well demonstrated that various metabolic risk factors related to MAFLD are associated with a systemic inflammatory response. Several studies have proven significant association between hsCRP and MAFLD risk factors. Frohlich et al. (38) reported that hsCRP was positively associated with BMI, TC, TG, and FPG but negatively associated with HDL-C levels. Timpson et al. (39) also demonstrated marked association between hsCRP and BMI, SBP, WHR, HDL-C, and TG, as well as HOMA-IR in British women. Moreover, higher hsCRP levels were found to be correlated with elevated ALT and AST in addition to higher BMI, FPG, TG, and LDL-C (30). Indeed, our findings confirmed that serum hsCRP levels were positively correlated with BMI, TG, ALT, AST, γ-GT, LnFINS, and LnHOMA-IR. Besides, the univariate logistic regression in our study showed that levels of hsCRP, BMI, γ-GT, and LnHOMA-IR were positively associated with the risk of MAFLD. Furthermore, hsCRP, BMI, γ-GT, LDL-C, and LnHOMA-IR were found to be significantly correlated with the risk of severe steatosis whereas hsCRP, BMI, TC, TG, and LnHOMA-IR were correlated with the risk of severe fibrosis. After adjusting these significant variables, these correlations remain significant. These findings raised the possibility that systemic inflammation is commonly accompanied by MAFLD and serum hsCRP could be an important clinical feature of MAFLD and its disease severity in addition to excess weight, dyslipidemia, elevated liver enzymes, and increased IR.

The underlying mechanism linking hsCRP to MAFLD may include the following points. One possibility might be that the liver is the main target organ of glucolipid metabolism regulation and is a key site of metabolic homeostasis and inflammation in obesity (40). Obesity causes a marked increase in the hepatic recruitment of macrophage, accompanied by the local production of inflammatory chemokines and cytokines that can induce IR in hepatocytes and ultimately promote hepatic steatosis (41). Furthermore, Koyama et al. (42) reviewed the close association among obesity, hepatic inflammation, and the development of NAFLD, which can progress to nonalcoholic steatohepatitis, fibrosis, and eventually cirrhosis due to sequential activation of inflammatory cascade (10). Another explanation is that overwhelming evidence suggests that IR is the main mediator linking inflammation to MAFLD. By inhibiting the key inflammatory signaling pathways including NF-κB and JNK pathways (43), as well as various proinflammatory signaling molecules and cytokines, the connection between obesity and IR can be blocked, finally causing significant tissue damage such as liver steatosis and fibrosis (44, 45). Due to that the roles of hsCRP in the MAFLD disease progression have been poorly understood, further investigation needs to be performed in large-scale populations.

There are some limitations to our work that must be acknowledged. First, our cross-sectional study might not reflect the causal relationship between serum hsCRP, MAFLD, and the severity of hepatic steatosis and fibrosis among Chinese obese patients. Second, the severity of hepatic steatosis and fibrosis in our study was assessed using noninvasive methods but not liver biopsy, which is well known to be the gold standard for the diagnosis of MAFLD. The reason is that these non-invasive techniques have been validated to be accurate and widely available in the general population (26). Third, other unmeasured confounding variables may exist. Finally, newly diagnosed MAFLD has not been widely applied in the real world. Despite these caveats, our findings provide a novel insight regarding the correlation of hsCRP with MAFLD among Chinese obese participants and raise the possibility that hsCRP might be used as a potential biomarker to monitor the disease severity in MAFLD. Future studies with many patients whose liver assessment is determined by non-invasive methods and MAFLD is diagnosed based on novel diagnostic criteria are warranted to further validate the findings from this study and identify their underlying mechanism.

## Conclusion

Serum hsCRP is an independent risk factor of MAFLD and positively associated with the severity of hepatic steatosis and fibrosis among the Chinese obese population even after adjusting for multiple confounding factors. These associations may be attributed to inflammation status and IR in addition to excess weight, dyslipidemia, and elevated liver enzymes. Further studies should focus on delving into the possible mechanism and its clinical significance.

## Data Availability Statement

The raw data supporting the conclusions of this article will be made available by the authors, without undue reservation.

## Ethics Statement

The studies involving human participants were reviewed and approved by the Research Ethics Review Committee of Shanghai Tenth People’s Hospital, School of Medicine, Tongji University, Shanghai, China. The patients/participants provided their written informed consent to participate in this study.

## Author Contributions

ZC and HD contributed to the first drafting of the manuscript. ZC, HD, MH, QC, and YH contributed to the data collection, statistical analysis, and interpretation of the data. QC revised this manuscript critically for important intellectual content. QS and BL contributed to the conception and design of this study, and critical revision of the manuscript, as well as approved the final version of the submitted manuscript. All authors read and approved the final manuscript.

## Funding

This work was financially supported by grants from the National Key R&D Program of China (No. 2018YFC1314100), Climbing Talent Program of the 10th People's Hospital affiliated to Tongji University (040120050), Clinical research funds for shanghai municipal health commission (202040170). the Shanghai Committee of Science and Technology of China (Nos. 18411951803 and 17DZ1910603), National Natural Science Foundation of China (No. 81970677 and 82170861), and the Shanghai Pujiang Program (Nos. 2019PJD040 and 2018PJD038).

## Conflict of Interest

The authors declare that the research was conducted in the absence of any commercial or financial relationships that could be construed as a potential conflict of interest.

## Publisher’s Note

All claims expressed in this article are solely those of the authors and do not necessarily represent those of their affiliated organizations, or those of the publisher, the editors and the reviewers. Any product that may be evaluated in this article, or claim that may be made by its manufacturer, is not guaranteed or endorsed by the publisher.
